# Supplementary material for: High-Throughput Preparation and High-Throughput Detection of Polymer-Dispersed Liquid Crystals Based on Ink-Jet Printing and Grayscale Value Analysis
Source: Molecules. 2023 Feb 28;28(5):2253. doi: 10.3390/molecules28052253 (PMC10005514; doi:10.3390/molecules28052253)
Supplement: Supplementary file 1 [file molecules-28-02253-s001.zip › molecules-2155521-supplementary.pdf]

The standard curves of ink solvents 15wt% PEGDA-600, 15wt% PETMP, 5wt% TMPTA and 30wt% LC solutions corresponding to CMYK channels are shown in the figure below.

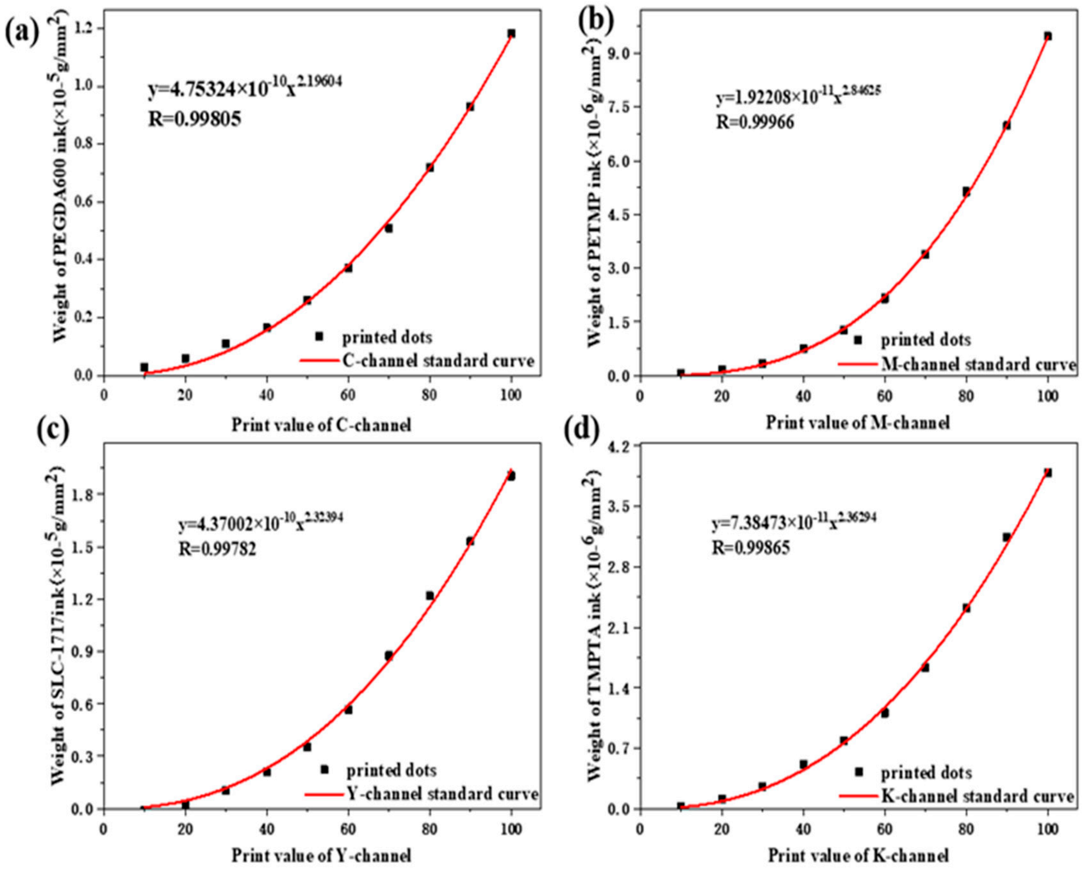

Figure S1. (**a**) Standard curve diagram of 15% PEGDA600 ink printed on the C channel; (**b**) Standard curve schematic of 15% PETMA ink printed on M channel; (**c**) Standard curve diagram of 30wt%SLC-1717 ink printed on Y channel; (**d**) Standard curve diagram of 5wt% TMPTA ink printed on K channel.

Table S1. Composition and voltage results of LC samples for high-throughput inkjet printing.

Sample No	PEGDA600(wt%)	PETMA(wt%)	TMPTA(wt%)	LC(wt%)	Voltage(V)
1	28	35	7	30	40.8
2	28	35	6	31	31.6
3	28	35	5	32	27.4
4	28	35	4	33	24
5	28	35	3	34	23
6	28	30	7	35	32.4
7	28	30	6	36	29.4
8	28	30	5	37	27
9	28	30	4	38	27
10	28	30	3	39	24
11	28	25	7	40	32.2
12	28	25	6	41	30.1
13	28	25	5	42	34.8
14	28	25	4	43	28
15	28	25	3	44	25.4
16	28	20	7	45	34.5
17	28	20	6	46	33.6
18	28	20	5	47	30.3
19	28	20	4	48	20.1
20	28	20	3	49	16.8
21	28	15	7	50	32.2
22	28	15	6	51	31.8
23	28	15	5	52	27.6
24	28	15	4	53	19.8
25	28	15	3	54	19.7
26	24	35	7	34	38.1
27	24	35	6	35	27.6
28	24	35	5	36	27
29	24	35	4	37	24.6
30	24	35	3	38	21.6
31	24	30	7	39	39.6
32	24	30	6	40	37.5
33	24	30	5	41	31.2
34	24	30	4	42	22.8
35	24	30	3	43	19.5
36	24	25	7	44	39
37	24	25	6	45	35.7
38	24	25	5	46	37.8
39	24	25	4	47	19.2
40	24	25	3	48	19.5
41	24	20	7	49	35.8
42	24	20	6	50	32.4
43	24	20	5	51	37.2
44	24	20	4	52	27.3
45	24	20	3	53	27
46	24	15	7	54	36.6
47	24	15	6	55	32.4
48	24	15	5	56	34.2
49	24	15	4	57	32.7
50	24	15	3	58	29.4
51	20	35	7	38	44.4
52	20	35	6	39	42.9
53	20	35	5	40	34.5
54	20	35	4	41	21.3
55	20	35	3	42	31.2
56	20	30	7	43	43.8

57	20	30	6	44	41.4
58	20	30	5	45	37.5
59	20	30	4	46	35.4
60	20	30	3	47	36
61	20	25	7	48	33.6
62	20	25	6	49	36.6
63	20	25	5	50	27.3
64	20	25	4	51	26.4
65	20	25	3	52	22.5
66	20	20	7	53	43.2
67	20	20	6	54	44.4
68	20	20	5	55	35.7
69	20	20	4	56	33
70	20	20	3	57	25.2
71	20	15	7	58	40.5
72	20	15	6	59	45.6
73	20	15	5	60	42.3
74	20	15	4	61	30.6
75	20	15	3	62	29.7
76	16	35	7	42	46.2
77	16	35	6	43	38.1
78	16	35	5	44	34.5
79	16	35	4	45	37.5
80	16	35	3	46	29.1
81	16	30	7	47	39.6
82	16	30	6	48	34.5
83	16	30	5	49	34.8
84	16	30	4	50	35.4
85	16	30	3	51	27.6
86	16	25	7	52	45.6
87	16	25	6	53	39.6
88	16	25	5	54	35.1
89	16	25	4	55	27
90	16	25	3	56	25.2
91	16	20	7	57	45.6
92	16	20	6	58	42
93	16	20	5	59	37.2
94	16	20	4	60	36.6
95	16	20	3	61	33
96	16	15	7	62	39.6
97	16	15	6	63	38.1
98	16	15	5	64	38.4
99	16	15	4	65	28.8
100	16	15	3	66	27.6
101	12	35	7	46	37.5
102	12	35	6	47	34.5
103	12	35	5	48	31.2
104	12	35	4	49	29.4
105	12	35	3	50	25.2
106	12	30	7	51	37.2
107	12	30	6	52	28.8
108	12	30	5	53	27.3
109	12	30	4	54	27.6
110	12	30	3	55	26.4
111	12	25	7	56	36.3
112	12	25	6	57	36.9
113	12	25	5	58	34.6
114	12	25	4	59	35.4
115	12	25	3	60	36.3

116	12	20	7	61	37.8
117	12	20	6	62	29.4
118	12	20	5	63	27
119	12	20	4	64	27
120	12	20	3	65	24.6
121	12	15	7	66	33
122	12	15	6	67	27
123	12	15	5	68	25.2
124	12	15	4	69	23.4
125	12	15	3	70	21.6
